# Biochemical Diversity and Nutraceutical Potential of Medicinal Plant-Based Herbal Teas from Southwestern Türkiye

**DOI:** 10.3390/plants15010125

**Published:** 2026-01-01

**Authors:** Halil Ibrahim Sagbas, Saban Kordali, Sena Sahin, Selçuk Küçükaydın, Elif Uyduran

**Affiliations:** 1 Department of Horticulture, Fethiye Faculty of Agriculture, Mugla Sitki Kocman University, Fethiye, Mugla 48300, Türkiye; 2 Department of Plant Protection, Fethiye Faculty of Agriculture, Mugla Sitki Kocman University, Fethiye, Mugla 48300, Türkiye; 3 Department of Medical Services and Techniques, Koycegiz Vocational School of Health Services, Mugla Sitki Kocman University, Koycegiz, Mugla 48800, Türkiye

**Keywords:** antioxidant activity, phenolic components, medicinal aromatic plant, herbal tea, ethanol extract

## Abstract

Medicinal and aromatic plants contain valuable natural compounds widely used in health, food, and cosmetics. This study compares the antioxidant capacities and phenolic compositions of tea and ethanol extracts from eight species naturally growing in Fethiye, Muğla, Türkiye. Antioxidant activity was assessed using the β-carotene bleaching method, 2,2-diphenyl-1-picrylhydrazyl (DPPH), 2,2′-azino-bis (3-ethylbenzothiazoline-6-sulfonic acid) (ABTS^+^), cupric reducing antioxidant capacity (CUPRAC), and metal chelating activity. Herbal teas generally showed stronger antioxidant activity than ethanol extracts. Rosemary tea had the highest activity (2.90 µg/mL), followed by lavender (11.30 µg/mL). In metal chelating, rosemary tea exhibited a half-maximal inhibitory concentration (IC_50_) of 9.22 µg/mL, close to ethylenediaminetetraacetic acid (EDTA). Phenolic profiling showed rosemary tea contained 30.74 mg/g rosmarinic acid and 0.74 mg/g quercetin. These results support the traditional use of southwestern Türkiye’s medicinal plants and emphasize the antioxidant potential of herbal teas. Integrating ethnobotanical knowledge with phytochemical data provides a basis for functional food development, crop improvement, and conservation of local plant genetic resources. Unlike previous studies focusing on single species or limited solvent comparisons, this research simultaneously evaluates both herbal tea and ethanol extracts of eight locally grown medicinal plants, offering a unique perspective on their comparative antioxidant and phenolic diversity.

## 1. Introduction

Medicinal and aromatic plants are widely used in the food, pharmaceutical, and cosmetic industries and have been utilized for various purposes throughout human history. Some of these plants are collected from their natural habitats, while others are cultivated for production. A significant portion of the plants used for therapeutic purposes are sourced from nature. The earliest references to herbal medicine date back to around 5000 BC in the Mesopotamian civilization, where approximately 250 herbal drugs were identified. Traditional herbal medicine, known by various names such as complementary medicine or natural therapy, is widely used, particularly in developing countries [1,2,3].

The prevalence of herbal medicine practices varies depending on the level of development of countries. In developing countries, approximately 80% of the population benefits from herbal products for therapeutic purposes. In regions such as Asia, Africa, and the Middle East, this proportion can reach up to 95% in some countries. In contrast, the use of medicinal plants is more limited in developed countries. For example, the rate is reported to be 48% in Australia, 40–50% in Germany, 42% in the United States, and 49% in France. However, the leading countries in the trade of medicinal plants are typically Germany, Japan, the United Kingdom, and the United States [4].

Many countries have enacted legal regulations regarding medicinal and aromatic plants and have introduced various directives in this field. A regulation related to medicinal and aromatic plants has been enforced in the Official Gazette published by the Presidency of the Republic of Türkiye [5]. The benefits of these plants, which have attracted significant attention from societies throughout history, have become the subject of scientific research in recent years and are supported by various academic studies [6,7,8,9].

For the widespread use of medicinal and aromatic plants, the geographic region where they grow must possess the ecological conditions that can provide these plants to people. In other words, in countries with high plant biodiversity, these plants can be utilized for various purposes. While there are approximately 12,500 plant species on the European continent, a total of 12,141 plant species and subspecies have been recorded in Türkiye [10]. This demonstrates that Türkiye is one of the countries with the richest plant biodiversity in Europe. According to data from the Ministry of Agriculture and Forestry of the Republic of Türkiye, 71.2% of the plant species in the country (8644 species) are not endemic, 25.9% (3148 species) are endemic, and 2.9% (349 species) are locally endemic. In the province of Muğla, where the study was conducted, a total of 2123 vascular plant species have been recorded. Of these, 83.8% (1780 species) are not endemic, 15.5% (329 species) are endemic, and 0.7% (14 species) are locally endemic [11]. These data clearly show the contribution of the Muğla province to Türkiye’s plant biodiversity.

Herbal teas obtained from medicinal and aromatic plants are aqueous herbal preparations with protective and therapeutic properties, made from a single plant drug or a combination of several drugs. Herbal teas are increasingly consumed not only for their delicious flavors and pleasant aromas but also for the health benefits they offer. Today, the plant species used for this purpose are quite diverse [12,13,14,15,16]. The consumption of foods, particularly plant teas rich in polyphenols, may play an important role in reducing the risk of various diseases associated with increased oxidative stress [17,18]. Considering the biological and pharmacological effects of the polyphenols absorbed through herbal teas, the positive effects of these compounds on health are of great importance. Studies have shown that polyphenols, due to their antioxidant properties, are associated with a reduction in the risks of coronary heart disease, stroke, and cancer. Additionally, some teas derived from certain plant species have been reported to offer not only antioxidant properties but also anti-inflammatory, anti-thrombotic, anti-hyperglycemic, and anti-mutagenic effects [19,20,21,22].

Previous studies on Mediterranean herbal teas have generally focused on single species or specific extraction techniques. However, comparative evaluations of multiple endemic and regionally important species under uniform ecological conditions are scarce. The present study fills this gap by simultaneously analyzing eight medicinal plants native to southwestern Türkiye, providing new insights into their antioxidant performance and phenolic composition.

The main aim of the study is to determine the phenolic component profiles of teas and ethanol extracts obtained from the leaves of certain medicinal and aromatic plants grown in the Fethiye district of Muğla province and to evaluate their antioxidant activities. The present research provides a scientific basis for the traditional use of these plants and highlights the importance of herbal teas as natural antioxidant sources. The findings offer valuable insights for the functional food and health supplement industries and may contribute to future research on natural antioxidants and their health benefits. Furthermore, the documentation of phytochemical diversity in medicinal plant species from southwestern Türkiye contributes to the conservation and sustainable use of regional plant genetic resources. By bridging traditional ethnobotanical knowledge with phytochemical profiling, this research supports the valorization of underutilized local taxa and provides a framework for their potential integration into crop improvement programs and germplasm conservation strategies.

## 2. Results and Discussion

### 2.1. Antioxidant Activity Determination

#### 2.1.1. Total Antioxidant Activity: β-Carotene Bleaching Method

When comparing the ethanol extracts and plant teas of the eight different plant species examined in the study, it was generally determined that the antioxidant activity values of the plant teas were higher. According to the total antioxidant activity analyses, the lowest total antioxidant activity values were found in the hawthorn ethanol extract at 73.91 µg/mL and the olive ethanol extract at 62.78 µg/mL. In contrast, the highest total antioxidant activity values were recorded in rosemary tea at 2.90 µg/mL, rosemary ethanol extract at 3.55 µg/mL, and eucalyptus tea at 8.97 µg/mL. In comparison with reference compounds, α-tocopherol (2.10 µg/mL) and BHA (Beta Hydroxycarboxylic Acid) (1.50 µg/mL) had lower total antioxidant activity values, with rosemary being the closest plant species to these values (Table 1).

#### 2.1.2. DPPH Free Radical Scavenging Activity Method

According to the DPPH free radical scavenging activity results, the lowest values were found in rosemary tea (4.76 µg/mL) and rosemary ethanol extract (6.12 µg/mL) samples. Compared to α-tocopherol (38.20 µg/mL), rosemary exhibited a significantly higher activity. Considering the reference value of BHA (19.50 µg/mL), rosemary (4.76 µg/mL), myrtle (16.80 µg/mL), lavender (17.61 µg/mL), myrrh (17.82 µg/mL), and eucalyptus (18.10 µg/mL), plant teas showed antioxidant activity similar to and competitive with BHA.

#### 2.1.3. ABTS^+^ Cation Radical Scavenging Activity Method

When examining the ABTS^+^ cation radical scavenging activity, the highest antioxidant activity was found in rosemary tea (3.42 µg/mL), followed by rosemary ethanol extract (4.80 µg/mL). These results demonstrate that rosemary has a strong effect in neutralizing the ABTS^+^ radical. In comparison with the reference values of BHA (12.70 µg/mL) and α-tocopherol (35.50 µg/mL), the samples of plant species such as rosemary, lavender, and bay laurel yielded results more consistent with BHA. Additionally, these plants showed much higher antioxidant activity compared to α-tocopherol.

#### 2.1.4. CUPRAC Method

According to the analysis performed by the CUPRAC method, rosemary tea (3.88 µg/mL) and rosemary ethanol extract (5.24 µg/mL) had the lowest values, representing strong antioxidant capacity. When compared with the reference compounds, BHA (25.40 µg/mL) and α-tocopherol (60.20 µg/mL), the samples of all plant species, except for olive and hawthorn, showed higher antioxidant activity in the CUPRAC analysis.

#### 2.1.5. Bipyridine Metal Chelating Activity

In antioxidant activity studies, EDTA (ethylenediaminetetraacetic acid) is a compound known for its ability to bind metal ions (chelating). EDTA exhibits antioxidant activity by preventing or reducing oxidation reactions catalyzed by free metal ions.

Our findings showed that EDTA (5.50 µg/mL) exhibited the highest metal chelation activity. Rosemary tea (9.22 µg/mL) and rosemary ethanol extract (14.71 µg/mL) were among the samples with the lowest values but showed the closest chelation activity to EDTA among all plant samples. These findings suggest that rosemary has a considerably higher capacity to bind metal ions compared to other plant species.

**Figure 1:**
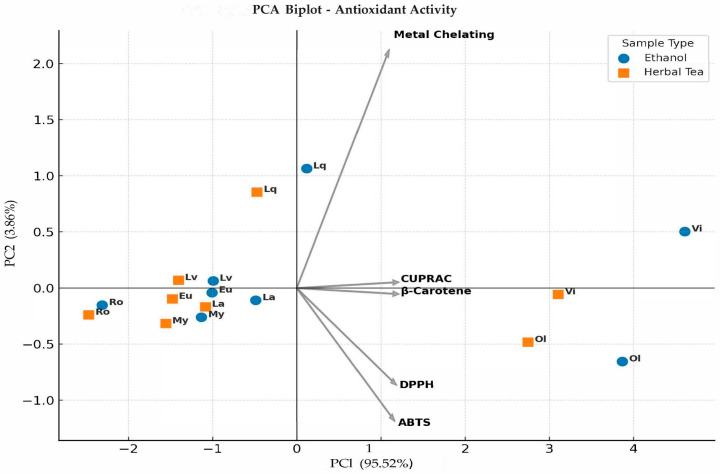
PCA biplot of antioxidant activity in ethanol and herbal tea extracts (Ro: Rosemary, La: Laurel, Lv: Lavender, Eu: Eucalyptus, Lq: Liquidambar, My: Myrtle, Ol: Olive, Vi: Vitex).

In the present study, separation along the first two principal components was primarily shaped by antioxidant assay parameters such as β-carotene bleaching, DPPH, ABTS, CUPRAC, and metal chelating activity (Figure 1). PC1 and PC2 explained 95.52% and 3.86% of the total variance, accounting for a cumulative 99.38%. The PCA biplot clearly distinguished ethanol extracts from herbal tea samples. PC1 mainly reflected overall antioxidant capacity. β-carotene and CUPRAC showed strong positive loadings, indicating their dominant role on this axis, whereas DPPH and ABTS loaded negatively, suggesting that samples positioned on the negative side had higher radical scavenging potential. Metal chelating activity contributed mostly to PC2 and separated samples according to their ion-binding capacity, particularly distinguishing liquidambar and vitex from the other species. Vitex and olive were positioned on the far right of PC1, showing strong β-carotene and CUPRAC activities. In contrast, rosemary and lavender clustered on the negative side of PC1, reflecting higher DPPH and ABTS efficiencies. Liquidambar differed along PC2 due to its elevated metal chelating capacity. Overall, these results indicate that antioxidant behavior in herbal teas and ethanol extracts is multidimensional and strongly assay-dependent. Moreover, the clear grouping of samples demonstrates the decisive influence of solvent type, with herbal teas generally exhibiting stronger reducing and radical scavenging activities than ethanol extracts.

In previous reports, rosemary has consistently shown strong antioxidant activity. For example, Kaygisiz [23] reported DPPH scavenging at 11.83 µg/mL and iron chelating activity at 62.01 µg/mL. Yazıcı et al. [24] found an IC50 of 2.75 μg/mL for rosemary essential oil, while Ranjbar Nedamani et al. [25] observed that rosemary extract exhibited higher DPPH scavenging than BHT up to 150 μg/mL. Although combinations with other extracts often produced antagonistic effects, pure rosemary extract retained strong activity. Malik and Upadhyay [26] showed that methanol extracts of rosemary effectively neutralized free radicals and suggested potential anti-aging applications. Rezanejad et al. [27] demonstrated that gamma radiation (30–40 kGy) enhanced DPPH and ABTS scavenging, although higher doses reduced Fe^2+^ chelation. Rosemary’s phytochemical profile also varies considerably: wild and cultivated populations differ in rosmarinic acid content [28]. Collectively, these studies show that rosemary’s antioxidant efficiency is largely driven by phenolic acids and flavonoids capable of electron transfer and hydrogen atom donation. This mechanistic framework supports the strong radical scavenging and metal chelating activities observed in our study.

In a study by Karadağ [29], the DPPH scavenging activity of laurel leaves was reported as 12.96 mg/mL, while the ABTS^+^ capacity and CUPRAC values were 1.03 mg/mL and 153.63 mg TE/g, respectively. Karataş et al. [30] similarly recorded a broad DPPH range (52.7–144.45 mg TE/g) and substantial ABTS^+^ capacity (74.64–115.08 mg TE/g). Kıvrak et al. [31] measured antioxidant activity in both ethanol extracts and teas of laurel leaves; ethanol extract values were 36.23 μg/mL (β-carotene), 43.74 μg/mL (ABTS^+^), and 129.10 μg/mL (DPPH), while herbal teas showed markedly higher antioxidant activity. These consistent observations indicate that the antioxidant potential of laurel largely depends on its phenolic and flavonoid constituents, including rutin, luteolin derivatives, and several hydroxybenzoic acids. Such compounds act primarily through electron donation and hydrogen atom transfer mechanisms, enabling both radical neutralization and metal-ion reduction. This mechanistic background aligns with the moderate yet reproducible antioxidant responses observed in the current study for both ethanol and tea extracts.

Karagözler et al. [32] reported strong antioxidant capacity in lavender, with CUPRAC values of 0.33 μg/mL in tea and 0.09 μg/mL in ethanol extracts, while DPPH activity varied substantially between extract types. Messaoud et al. [33] also demonstrated broad variability, noting DPPH values ranging from 15.8 to 34.2 μg/mL and FRAP activities from 224.1 to 505.4 μg/mL. These variations largely reflect differences in extraction polarity and phenological stage. Lavender’s antioxidant efficiency is closely linked to rosmarinic acid derivatives and flavones such as luteolin and apigenin. These molecules stabilize reactive oxygen species through resonance delocalization and efficient electron redistribution within their aromatic ring structures. This structural behavior enhances both hydrogen atom transfer and electron-transfer pathways. Consequently, the contrasting antioxidant levels observed between aqueous and ethanol extracts in the present study reflect the selective extraction of polar phenolic constituents, particularly rosmarinic acid, which is more efficiently solubilized in herbal tea preparations.

Eucalyptus extracts also exhibited notable antioxidant activity. In an Iranian study [34], DPPH scavenging values ranged widely (0.50–57.10 μg/mL), illustrating high chemical variability. Bhuyan et al. [35] found exceptionally strong antioxidant responses in plant teas, with ABTS^+^, DPPH, and CUPRAC values reaching 525.67 mg TE/g, 378.61 mg TE/g, and 607.43 mg TE/g, respectively. These effects can be attributed to eucalyptus’ enrichment in polyhydroxylated flavonols such as myricetin and quercetin, compounds with strong metal-ion chelating and peroxyl radical-neutralizing capacities. Their multiple hydroxyl groups confer high reactivity toward electron-deficient radicals. Moreover, the co-occurrence of phenolic acids enhances redox cycling, allowing these flavonols to regenerate primary antioxidants after oxidation. This mechanistic interplay is consistent with our findings, in which eucalyptus teas generally outperformed ethanol extracts, reflecting the improved extraction of polar flavonols in aqueous preparations.

Wang et al. [36] evaluated ethanol, herbal tea, and acetone extracts obtained from the leaves of liquidambar in China and reported that ethanol extracts exhibited the highest antioxidant activity. Similarly, Ulusoy et al. [37] analyzed water extracts of liquidambar leaves from the Muğla region, obtaining CUPRAC, FRAP, DPPH, and ABTS^+^ values of 49.25 mmol/g TE, 39.83 µmol Fe/g, 80.34 µg/mL, and 51.20 µg/mL, respectively. These findings emphasize the strong influence of extraction polarity and solvent affinity on the antioxidant performance of Liquidambar species. The higher efficiency of ethanol extracts indicates that moderately polar solvents can release a broader spectrum of bioactive compounds, suggesting that antioxidant capacity is shaped not only by the total phenolic content but also by solvent-dependent differences in molecular solubility and extractability. Overall, previous studies consistently demonstrate that many medicinal and aromatic plants show high antioxidant potential, and herbal teas in particular often provide strong radical scavenging activity due to the enhanced extraction of polar phenolics.

### 2.2. Identification of Phenolic Compounds

When comparing ethanol and herbal tea extracts of the eight studied species, herbal teas generally contained higher levels of phenolic compounds. Gallic acid was not detected in rosemary, laurel, lavender, and olive, whereas vitex ethanol extract contained the lowest amount (0.11 mg/g) and liquidambar tea the highest (5.10 mg/g). The absence of gallic acid in several species suggests limited synthesis or accumulation capacity and aligns with literature indicating substantial species- and solvent-dependent variation. Protocatechuic acid was also absent in laurel and olive. Its lowest level occurred in rosemary ethanol extract (0.10 mg/g), while the highest was found in vitex tea (17.68 mg/g), supporting the notion that its distribution is strongly species-specific.

Catechin was absent in nearly all species except laurel tea (0.17 mg/g), indicating that this compound is not commonly present in the examined taxa. Pyrogallol was detected only in laurel tea (0.15 mg/g), liquidambar ethanol extract (0.32 mg/g), and liquidambar tea (0.28 mg/g). Chlorogenic acid was absent in laurel, liquidambar, myrtle, and olive, whereas eucalyptus ethanol extract and lavender tea showed higher levels (0.13–5.80 mg/g). p-Hydroxybenzoic acid appeared only in a limited number of species, with rosemary ethanol extract presenting the lowest content (0.13 mg/g) and lavender ethanol extract the highest (0.74 mg/g). The coumarin derivative 6,7-dihydroxycoumarin was found only in laurel extracts (0.11 mg/g in ethanol; 0.24 mg/g in tea).

Caffeic acid and quercetin were among the most abundant phenolics. Caffeic acid ranged from 0.18 mg/g (myrtle tea) to 13.20 mg/g (lavender tea). Quercetin levels varied widely, from 0.10 mg/g (lavender ethanol) to 13.56 mg/g (laurel tea). Several phenolics, including 3-hydroxybenzoic acid, taxifolin, hesperidin, and vanillin, were not detected in any species. Syringic acid appeared only in laurel, rosemary tea, and myrtle, ranging from 0.11 to 0.27 mg/g. p-Coumaric acid was absent in rosemary ethanol extract, eucalyptus, myrtle, olive, and vitex but reached up to 3.65 mg/g in other samples. Ferulic acid was not detected in eucalyptus and liquidambar.

Coumarin was identified exclusively in olive, at 2.20 mg/g in tea and 2.71 mg/g in ethanol extract. Rutin was absent in myrtle, vitex, laurel, and lavender but present in other species (0.11–1.57 mg/g). Ellagic acid was not detected in liquidambar, olive, vitex, laurel, or lavender; its lowest level occurred in rosemary tea (0.10 mg/g), and the highest in myrtle tea (4.18 mg/g), reflecting species-specific accumulation patterns. Rosmarinic acid was absent in laurel, eucalyptus, liquidambar, myrtle, and vitex but reached its highest level in rosemary tea (30.74 mg/g), consistent with the well-documented enrichment of this compound in rosemary. Myricetin appeared only in liquidambar, eucalyptus, and myrtle (3.17–11.32 mg/g).

Trans-cinnamic acid was undetected in eucalyptus, myrtle, vitex, and olive, while laurel ethanol extract showed the lowest amount (0.10 mg/g) and liquidambar tea the highest (3.95 mg/g). Luteolin was absent in laurel, lavender, liquidambar, and myrtle but ranged from 0.17 mg/g (rosemary ethanol) to 4.10 mg/g (olive tea). Kaempferol occurred only in laurel and myrtle (2.14–5.39 mg/g). Apigenin was detected in lavender and eucalyptus (0.07–0.27 mg/g). Chrysin was absent in lavender, eucalyptus, styrax, myrtle, and olive; rosemary ethanol extract contained the lowest amount (0.15 mg/g), and laurel tea the highest (1.18 mg/g).

Overall, these findings demonstrate pronounced species-specific and solvent-dependent variation in phenolic composition. Differences in compound polarity, biosynthetic pathways, and tissue localization likely underpin these patterns. The data met ANOVA assumptions of normality and homogeneity, ensuring the reliability of statistical comparisons.

Mena et al. [38] reported several phenolic constituents in rosemary, including caffeic acid (0.03 mg/mL), p-coumaric acid (0.01 mg/mL), luteolin (0.14 mg/mL), apigenin (0.55 mg/mL), hesperidin (0.26 mg/mL), rosmarinic acid (0.12 mg/mL), and rutin (0.04 mg/mL). Vallverdú-Queralt et al. [39] also identified a broad phenolic spectrum in rosemary water–alcohol extracts, with notable levels of caffeic acid (12.58 μg/mL DW), p-coumaric acid (5.57 μg/mL DW), p-hydroxybenzoic acid (15.16 μg/mL DW), protocatechuic acid (8.42 μg/mL DW), and especially rosmarinic acid (156.9 μg/mL DW). Catechin and epicatechin were not detected. In the same study, bay leaf extracts contained moderate amounts of caffeic acid, ferulic acid, p-coumaric acid, p-hydroxybenzoic acid, protocatechuic acid, rosmarinic acid, and syringic acid, whereas catechin, epicatechin, and quercetin remained undetectable.

Peixoto et al. [40] analyzed rosemary herbal teas from four commercial sources and observed measurable amounts of protocatechuic acid (0.33–0.46 μg/mL), 4-hydroxybenzoic acid (0.17–0.26 μg/mL), caffeic acid (0.09–0.46 μg/mL), dihydroxycoumarin (0.06 μg/mL), p-coumaric acid (0.13–0.19 μg/mL), luteolin (0.59–1.16 μg/mL), apigenin (0.05–0.14 μg/mL), rosmarinic acid (42.03–47.54 μg/mL), and hesperidin (0.71–0.97 μg/mL). The high levels of rosmarinic acid, together with substantial quantities of caffeic acid and luteolin, indicate that rosemary’s antioxidant activity is driven by multiple hydrogen-donating phenolic structures acting synergistically. Rosmarinic acid is especially effective because its extended π-electron system stabilizes phenoxyl radicals and supports both hydrogen atom transfer (HAT) and electron-transfer (ET) mechanisms. Luteolin and apigenin derivatives likely complement this activity by functioning as secondary redox recyclers that regenerate oxidized cinnamic acid derivatives during radical quenching. The combined presence of hydroxycinnamic acids and flavone scaffolds thus provides a mechanistic explanation for the strong DPPH and ABTS responses typical of rosemary infusions, confirming that the plant’s high antioxidant capacity is structurally determined rather than merely a consequence of concentration differences.

Čulina et al. [41] analyzed laurel leaves using different extraction methods and reported rutin levels of 76.92–114.43 mg/100 g DW, catechin levels of 2.44–2.63 mg/100 g DW, luteolin levels of 17.60–26.22 mg/100 g DW, 4-hydroxybenzoic acid levels of 6.01–7.89 mg/100 g DW, and protocatechuic acid levels of 11.36–37.58 mg/100 g DW. However, kaempferol, rosmarinic acid, myricetin, quercetin, apigenin, gallic acid, vanillin, caffeic acid, chlorogenic acid, ferulic acid, and p-coumaric acid were not detected in these samples. In a comprehensive review, Konovalov and Alieva [42] reported similar trends, identifying trace concentrations of kaempferol-3-O-α-L-(3″,4″-di-E-p-coumaroyl) rhamnoside (0.00027%), luteolin (0.45%), quercetin (0.0152%), rutin (0.0929%), gallic acid and vanillin (1.40%), rosmarinic acid (0.02%), catechin (0.00916%), and epicatechin (1.29%). Collectively, these findings suggest that laurel’s antioxidant capacity is driven primarily by a narrow group of dominant phenolics, particularly rutin and luteolin, while most other compounds occur at trace or non-detectable levels. This restricted phenolic diversity provides a mechanistic explanation for the generally moderate antioxidant activity observed in laurel compared to species with richer phenolic profiles.

Sriti et al. [43] investigated lavender leaves collected during pre-flowering and flowering stages and demonstrated a strong dependency on solvent and phenophase. Caffeic acid was absent in ethanol and water extracts but ranged from 0.47 to 23.12 mg/g in methanol extracts. Gallic acid appeared only in the water extract during the flowering stage (0.029 mg/g). Syringic acid was restricted to methanol extracts (0.69–1.14 mg/g) and was more abundant in leaves than flowers, contradicting common assumptions about floral enrichment. p-Coumaric acid was detected across all extraction types (0.10–0.92 mg/g). Rosmarinic acid appeared only in water extracts of flowering-stage leaves (0.12 mg/g). Ferulic acid was found in all samples except the pre-flowering water extract (0.048–8.34 mg/g). Several phenolics, including ellagic acid, chlorogenic acid, myricetin, and luteolin, were present exclusively in flowering-stage water extracts at low concentrations (0.007–0.14 mg/g). Coumarin showed the highest extraction efficiency in methanol (3.08–5.21 mg/g) but was also detected at 0.18 mg/g in flowering-stage water extracts. These patterns highlight the pronounced extraction- and stage-specific nature of lavender’s phenolic composition. Because many key compounds appear only under certain solvent × developmental-stage combinations, lavender exhibits highly variable antioxidant responses, reflecting a chemical profile that shifts dynamically with both polarity and phenophase.

Mykhailenko et al. [44] examined methanolic extracts of flowering-stage lavender leaves from Ukraine and reported the following phenolic levels: caffeic acid (0.37–3.84 mg/g), p-coumaric acid (0.07 mg/g), rosmarinic acid (0.21–1.16 mg/g), ferulic acid (0.60 mg/g), chlorogenic acid (0.24–4.62 mg/g), vanillin (5.99–11.20 mg/g), gallic acid (0.06–0.12 mg/g), luteolin (0.02–0.11 mg/g), and apigenin (0.01–0.28 mg/g). In Türkiye, Karan [45] found trace levels of catechin, gallic acid, chlorogenic acid, rutin, hesperidin, apigenin, quercetin, and kaempferol, while protocatechuic acid (1.49 ppm), caffeic acid (27.85 ppm), vanillin (2.30 ppm), p-coumaric acid (3.25 ppm), ferulic acid (6.43 ppm), and rosmarinic acid (80.89 ppm) were also detected. Cáceres-Cevallos et al. [46] reported rosmarinic acid (1.0–4.5 mg/g DW), caffeic acid (0.04–0.2 mg/g DW), p-coumaric acid glucoside (0.3–1.7 mg/g DW), ferulic acid (0.2–0.9 mg/g DW), luteolin (0.1–1.6 mg/g DW), apigenin (0.02–0.1 mg/g DW), and o-coumaric acid (0.3–2.8 mg/g DW) in lavender ecotypes from Spain. Overall, these studies show that lavender’s phenolic profile varies substantially among regions and extraction methods, with rosmarinic acid, caffeic acid, and ferulic acid consistently forming its principal antioxidant backbone.

Dezsi et al. [47] reported that ethanol extracts of Australian eucalyptus leaves contained very low chlorogenic acid (<0.02 μg/g DW) and no detectable p-coumaric acid but were enriched in flavonols such as rutin (48.65 μg/g DW), myricetin (92.34 μg/g DW), quercetin (2.01 μg/g DW), luteolin (34.40 μg/g DW), and apigenin (2.85 μg/g DW). Ashraf et al. [48] demonstrated strong solvent-dependent variation in Pakistani eucalyptus: methanol extracts yielded gallic acid (5.86 μg/mL), vanillin (4.53 μg/mL), syringic acid (1.75 μg/mL), p-hydroxybenzoic acid (2.91 μg/mL), catechin (2.91 μg/mL), and quercetin (0.69 μg/mL); chloroform extracts contained gallic acid (2.21 μg/mL) and p-coumaric acid (0.12 μg/mL); and hexane extracts showed lower levels of gallic acid (0.42 μg/mL). Collectively, these findings indicate that eucalyptus is consistently dominated by flavonols, particularly myricetin, rutin, and luteolin, while hydroxycinnamic acids are generally scarce. This flavonol-oriented profile explains the tendency of eucalyptus to cluster with high-antioxidant species in comparative phenolic analyses.

Özbek and Bilek [49] conducted a study on the bioactive components of liquidambar leaves and found the following concentrations: gallic acid (3.26 mg/g), protocatechuic acid (12.23 mg/g), catechin (1.62 mg/g), chlorogenic acid (0.43 mg/g), caffeic acid (1.27 mg/g), p-coumaric acid (0.30 mg/g), ferulic acid (0.81 mg/g), quercetin (0.17 mg/g), and kaempferol (0.006 mg/g). Similarly, Taş Küçükaydın [50] analyzed the phenolic components of liquidambar leaf extracts obtained from the sycamore forests in Muğla, Turkey. In this work, pyrocatechol, p-hydroxybenzoic acid, taxifolin, ellagic acid, and rosmarinic acid were not detected in the sycamore samples. However, the study found the following concentrations: gallic acid (19.83 mg/g), protocatechuic acid (4.48 mg/g), catechin (0.91 mg/g), caffeic acid (0.75 mg/g), vanillin (3.44 mg/g), p-coumaric acid (4.79 mg/g), rutin (4.33 mg/g), myricetin (37.50 mg/g), quercetin (6.36 mg/g), trans-cinnamic acid (9.65 mg/g), and kaempferol (1.05 mg/g). These data indicate that liquidambar does not have a fixed phenolic pattern and that its dominant compounds can shift across populations. The large variation, particularly in gallic acid, myricetin and p-coumaric acid, suggests that liquidambar is a chemically variable taxon, which may explain the wide range of antioxidant responses reported in different studies.

Čulina et al. [41] reported that myrtle leaves contained high levels of myricetin (105.49–224.91 mg/100 g DW), rutin (4.14–4.87 mg/100 g DW), quercetin (4.31–4.61 mg/100 g DW), catechin (0.51–1.90 mg/100 g DW), luteolin (2.97–10.63 mg/100 g DW), gallic acid (11.57–15.70 mg/100 g DW), and caffeic acid (3.66–32.34 mg/100 g DW), while kaempferol, apigenin, 4-hydroxybenzoic acid, vanillin, chlorogenic acid, ferulic acid, p-coumaric acid, and rosmarinic acid were not detected. Değirmencioğlu et al. [51] found that drying strongly altered myrtle’s phenolic profile: gallic acid increased from 6.53 to 46.64 mg/kg (fresh) to 67.43–352.30 mg/kg (dried), and vanillin rose from 18.00 to 167.59 mg/kg to 20.78–1156.80 mg/kg. In contrast, caffeic acid decreased from 14.49–248.76 mg/kg to 3.28–28.24 mg/kg, and chlorogenic acid also declined. Myricetin ranged from 56.33 to 141.45 mg/kg (fresh) and 49.44–237.56 mg/kg (dried), while quercetin, ferulic acid, syringic acid, hesperidin, and catechin all showed variable responses to drying. These results demonstrate that myrtle’s phenolic composition is highly sensitive to post-harvest processing. Increases in some compounds and reductions in others reflect differences in thermal stability and oxidation sensitivity, underscoring the need for optimized drying conditions to preserve key bioactive components.

**Figure 2:**
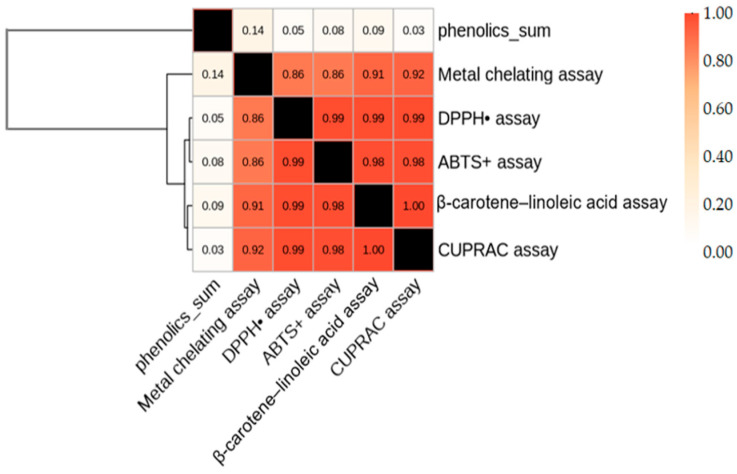
Heatmap of correlations between phenolic compounds and antioxidant capacity.

To examine the relationship between phenolic composition and antioxidant activity, Pearson correlation analysis combined with hierarchical clustering was applied. The heatmap (Figure 2) illustrates the strength and direction of correlations, while the dendrogram groups variables based on similarity. Most phenolic compounds showed strong positive associations with antioxidant assays, with correlation coefficients ranging from +0.65 to +0.99, indicating that higher phenolic levels substantially enhance the antioxidant capacity of the teas. DPPH, ABTS, β-carotene–linoleic acid, and CUPRAC clustered tightly together (r ≈ 0.98–1.00), reflecting their shared reliance on radical scavenging mechanisms. In contrast, metal chelating activity formed a separate branch and exhibited lower correlations with these assays (r ≈ 0.86–0.92), consistent with its distinct mechanism involving ion-binding rather than hydrogen- or electron-transfer reactions.

From a phenolic composition perspective, the hierarchical cluster analysis identified clear subgroups of compounds with different contributions to antioxidant activity. The first major cluster, rosmarinic acid, quercetin, ferulic acid, and caffeic acid, showed strong positive correlations with DPPH, ABTS, and CUPRAC (r > 0.85), indicating that these compounds act as primary determinants of total antioxidant capacity (Table 2). A second, smaller cluster containing ellagic acid, p-hydroxybenzoic acid, and syringic acid exhibited weaker associations (r < 0.60) and clustered farther from the antioxidant assays, suggesting a more limited or indirect role. Overall, the hierarchical structure highlights mechanistic differences among phenolics: compounds positioned close to radical scavenging tests likely function as direct hydrogen or electron donors, whereas more distant phenolics may contribute through secondary stabilization or metal chelating pathways. These findings demonstrate that antioxidant behavior in herbal teas is driven mainly by high-activity phenolics and that clustering effectively groups compounds with similar chemical and functional antioxidant characteristics.

Mechi et al. [52] analyzed olive leaf extracts obtained with hexane, diethyl ether, ethyl acetate, methanol, and water and found that methanol yielded the highest levels of phenolics, followed by ethyl acetate and diethyl ether, while hexane contained the lowest. Detected compounds included quercetin (50.3–2980.6 mg/g), gallic acid (1.1–6.4 mg/100 g), vanillin (0.3–3.3 mg/100 g), caffeic acid (11.1–532.9 mg/100 g), p-coumaric acid (0.8–3.8 mg/100 g), and chlorogenic acid (25.9–117.7 mg/100 g). Borghini et al. [53] similarly reported that methanol extracts of 29 olive leaf samples from Tuscany contained luteolin (0.18–2.60 g/kg DW), apigenin (0.08–7.95 g/kg DW), and rutin (0.26–1.47 g/kg DW), noting that phenolic fingerprints allowed reliable discrimination among olive cultivars. Siamandoura and Tzia [54] found that water extracts lacked vanillin, rutin, and luteolin and contained low caffeic acid (0.01 mg/g DW), whereas ethanol extracts yielded higher caffeic acid (0.03 mg/g DW), vanillin (0.39 mg/g DW), and rutin (5.91 mg/g DW), although luteolin remained undetected. These findings collectively highlight strong solvent-dependent extraction patterns and indicate that phenolic profiles can serve as potential markers for olive variety differentiation.

Kavaz et al. [55] demonstrated that vitex seeds contain substantial levels of quercetin (198.75 μg/L), fumaric acid (274.58 μg/L), caffeic acid (168.68 μg/L), ellagic acid (50.46 μg/L), luteolin (924.41 μg/L), and kaempferol (81.97 μg/L), while myricetin, gallic acid, and protocatechuic acid were not detected. These results indicate a phenolic-rich profile dominated by flavonoids. Berrani et al. [56] further reported large compositional differences among vitex organs grown in Morocco. In the seeds, chlorogenic acid (122,900 μg/kg), caffeic acid (44,277.2 μg/kg), luteolin (40,212.2 μg/kg), vanillin acid (19,176 μg/kg), p-hydroxybenzoic acid (22,771 μg/kg), quercetin (300.27 μg/kg), and pyrogallol (546.54 μg/kg) were prominent, while catechin, rutin, syringic acid, and ferulic acid appeared at lower levels. Their study showed that roots and stems were richer in phenolic acids, while leaves and flowers exhibited a more balanced mixture of phenolic acids and flavonoids. Seeds, in contrast, were especially enriched in chlorogenic acid and luteolin. This organ-specific variation suggests that different parts of the vitex plant may support distinct biological activities and application potentials.

In conclusion, these studies show that plant phenolic components vary significantly depending on the solvent used and the plant’s different organs. These findings provide valuable information for optimizing extraction processes and enhancing the bioavailability of plant phenolic compounds. The use of different solvents in the extraction process affects the quantity and diversity of these compounds, allowing for the development of optimization strategies that could help maximize the health benefits of these compounds.

## 3. Materials and Methods

### 3.1. Plant Material

The material for the conducted study consists of one genotype from each of eight different medicinal and aromatic plant species naturally growing on the campus of Fethiye Agriculture Faculty, Muğla Sitki Kocman University (36°38′27″ N, 29°13′33″ E). For each species (seeds for vitex and leaves for the others), samples were collected from five healthy individuals of similar age and ecological conditions. To minimize intraspecific variation and obtain a representative sample for each species, the collected materials were pooled and homogenized into a single composite sample, which was subsequently used for all analyses. All biochemical and antioxidant assays were performed in three independent replicates (*n* = 3) from the homogenized pooled samples.

**Figure 3:**
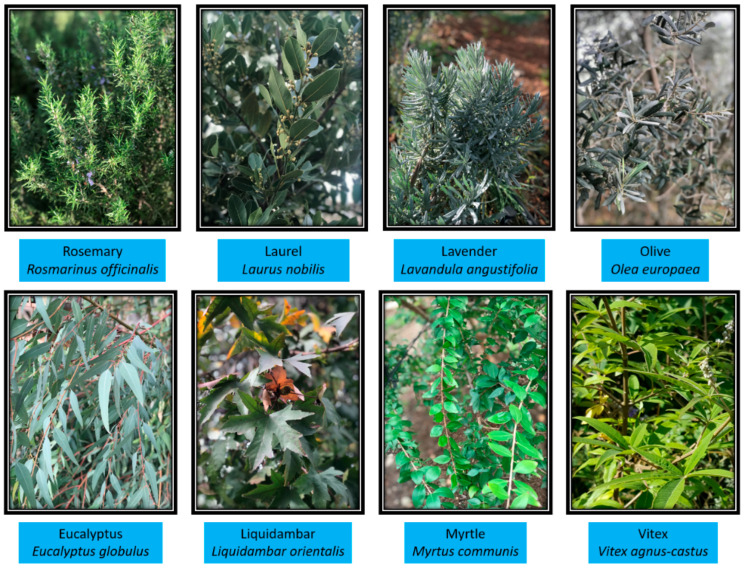
Images of the plant species used in the study (original).

The plants used in the study include myrtle (*Myrtus communis* L.), liquidambar (*Liquidambar orientalis* Mill.), eucalyptus (*Eucalyptus globulus* Labill.), olive (*Olea europaea* L.), lavender (*Lavandula angustifolia* Mill.), rosemary (*Rosmarinus officinalis* L.), vitex (*Vitex agnus-castus* L.), and laurel (*Laurus nobilis* L.) (Figure 3). The taxonomic identification of all plant species was performed by Prof. Dr. Saban Kordali, an expert in herbology.

The plant species included in this study were selected based on their natural occurrence and traditional importance in the western and southern regions of Türkiye. These species are either native to these areas or represent exotic taxa that were introduced to the region many years ago and have successfully adapted to local ecological conditions through historical acclimatization processes. Furthermore, the selected plants have been utilized by local communities for centuries for multiple purposes. They are commonly used as food ingredients—particularly as herbal teas and spices—as well as valuable sources of essential oils employed in traditional medicine, perfumery, and other aromatic industries. Although some of the investigated species may not currently be among the most globally popular herbal teas, they possess strong local and cultural significance in southwestern Türkiye, where the consumption of herbal teas remains a deeply rooted and widespread tradition.

The secondary metabolite content of plants can vary depending on various environmental and physiological factors such as their age, altitude, seasonal conditions, and daily circadian rhythms [57,58,59]. In this context, the plants used in the current study were collected during the same phenological period (full flowering stage) and in the early hours of the day in 2024. To ensure standardization, samples were collected on specific dates corresponding to the full flowering stage of each species: liquidambar on 16 April, laurel on 29 April, rosemary on 10 May, olive on 14 May, myrtle on 1 June, lavender on 7 June, eucalyptus on 22 October, and vitex on 13 November. The forms of use of medicinal and aromatic plants vary according to their species. Except for the vitex tree, the leaves of the plants are generally consumed as tea, while the seeds of the vitex tree are used. Therefore, in the study, leaf samples were collected from myrtle, liquidambar, eucalyptus, olive, lavender, rosemary, and laurel, whereas seed samples were collected from the vitex tree. Additionally, the plants were selected with similar ages, which was considered an important criterion for the reliability of the study. Plants estimated to be between 5 and 10 years old were preferred.

Although voucher specimens were not deposited in a herbarium, the plant materials were collected from naturally growing individuals located within the campus of Fethiye Agriculture Faculty, Muğla Sitki Kocman University. The exact collection sites and GPS coordinates were recorded, allowing the same plants to be re-sampled in the future if verification or additional analyses are needed.

### 3.2. Obtaining Teas and Extracts

Plant samples were collected during the vegetation period. Then all samples (plant leaves and vitex seeds) were oven-dried in a ventilated laboratory oven at 40 ± 2 °C until a constant weight was reached (approximately 48–72 h). This relatively low temperature was selected to minimize thermal degradation of phenolic compounds and preserve antioxidant activity. After drying, samples were ground using a laboratory mill, placed in airtight glass containers, and stored at 4 °C in the dark until analyses were performed.

In the present study, ethanol was selected as the extraction solvent based on its superior efficiency, safety, and compatibility with food and nutraceutical applications, as supported by a recent study by Bodoira and Maestri [60], which comprehensively evaluated the extraction efficiency of different solvents, including ethanol, methanol, and acetone, and found ethanol to be the most effective in terms of phenolic yield. Two different methods were then applied to obtain both tea and ethanol (70:30, *v*/*v*) extracts from the dried plant materials. For the preparation of plant teas, the method used by Gölükçü et al. [61] was followed, and the teas were prepared in triplicate. From each plant species, 1.47 g of dried plant material was weighed using a precision scale and then boiled in 200 mL of water at 100 °C for 1 min. After boiling, the samples were removed from the heat source and left to steep for 4 min. Following the steeping process, the plant material was filtered and transferred into sterile containers.

For the preparation of ethanol extracts, the method used by Dönmez et al. [62] was adopted. First, the dried plant materials were ground, and 50 g of each plant species was weighed using a precision scale and transferred into 400 mL sterile jars. Ethanol was added to the jars, and the mixtures were stored in a cool, dry, and shaded environment. During the extraction process, the mixtures were shaken twice daily to prevent sedimentation and left to stand for at least 48 h. After the completion of the process, the extracts were filtered to separate them from the plant material. Finally, the liquid extracts obtained were separated from the solvent using a rotary evaporator and transferred into sterile containers. During solvent evaporation, the extracts were concentrated under reduced pressure at 40 °C using a rotary evaporator (Heidolph Scientific Products Company, Schwabach, Germany) to prevent thermal degradation and minimize solvent loss. Also, the extraction yield of the samples was 4.80% in the olive–herbal tea samples, while the highest value was observed in the rosemary ethanol extract at 12.35% (Table S1).

The validity of the HPLC-DAD method was clearly demonstrated. Calibration curves were constructed for each phenolic standard, and the corresponding calibration equations, regression coefficients (R^2^), linear ranges, limits of detection (LOD), and limits of quantification (LOQ) are presented in Table S2. Nearly all R^2^ values were above 0.999, indicating excellent linearity for the analytes. Prior to quantification, all ethanol extracts were normalized to a fixed concentration of 10 mg/mL before HPLC injection, ensuring comparability among samples and keeping the analyte responses within the linear dynamic range of the method. Therefore, all quantitative data were generated on the same concentration basis.

### 3.3. Determination of Antioxidant Activity

#### 3.3.1. Total Antioxidant Activity: β-Carotene Bleaching 

The total antioxidant activity of the plant samples was determined using the β-carotene–linoleic acid method. In this method, the inhibition of conjugated diene hydroperoxides formed by the oxidation of linoleic acid and the bleaching of β-carotene color were measured [63]. To 40 µL of samples at different concentrations, 160 µL of β-carotene solution was added, and the initial absorbance values were measured at 470 nm using a 96-well microplate reader. The samples were incubated at 45 °C, and the incubation continued until the color of β-carotene disappeared (approximately 120 min). After the incubation period, absorbance measurements were taken again, and the bleaching rate (R) of β-carotene was calculated according to the following equations.

(1) $$R=\frac{ln\frac{a}{b}}{t}$$

where ln = natural log, *a* = absorbance at time zero, *b* = absorbance at time *t* (120 min).

(2) $$Antioxidant\;Activity\left(Inhibition\;\%\right)=\frac{R_{control}-R_{sample}}{R_{control}}\times 100$$

R_control_: the bleaching rate of control, R_sample_: the bleaching rate of sample.

#### 3.3.2. DPPH Free Radical Scavenging Activity 

The DPPH free radical scavenging activity of the samples was determined according to the method described by Djebili et al. [64]. In this method, the bleaching of the DPPH solution was measured to determine the free radical scavenging capacity. To 40 µL of samples at different concentrations, 160 µL of 0.4 mM DPPH solution was added, and the mixture was incubated at room temperature in a dark environment for 30 min. Subsequently, absorbance values were measured at 517 nm using a 96-well microplate reader, and the free radical scavenging activity was calculated according to the following equation.

(3) $$DPPH\;Scavenging\;Effect\left(\%\right)=\frac{A_{control}-A_{sample}}{A_{control}}\times 100$$

A_control_: the absorbance of the control, A_sample_: the absorbance of the sample.

#### 3.3.3. ABTS^+^ Cation Radical Scavenging Activity 

In the study, the ABTS^+^ cation radical was used to determine the free radical scavenging activity of the samples [65]. In this method, the bleaching of the ABTS^+^ solution was measured to evaluate the radical scavenging capacity. To 40 µL of samples at different concentrations, 160 µL of ABTS^+^ solution was added, and the mixture was incubated at room temperature for 10 min. Absorbance values were then read at 734 nm using a 96-well microplate reader, and the results were calculated as ABTS^+^ cation radical scavenging activity according to the following equation.

(4) $${ABTS}^{+}\;Scavenging\;Effect\;(\%)=\frac{A_{control}-A_{sample}}{A_{control}}\times 100$$

A_control_: the absorbance of the control, A_sample_: the absorbance of the sample.

#### 3.3.4. CUPRAC Method (Copper Reducing Antioxidant Capacity)

The CUPRAC method was used to determine the total antioxidant activity of the samples, measuring the capacity of antioxidants to reduce copper ions (Cu^2+^) [66]. For the analysis, 100 μL of appropriately diluted samples was taken, and 1 mL of 10^−2^ mM copper (II) chloride (CuCl_2_·2H_2_O) solution, 1 mL of 7.5 × 10^−3^ neocuproine solution, 1 mL of 1 M ammonium acetate buffer solution (pH: 7), and 1 mL of distilled water were added sequentially. The mixture was incubated at room temperature for 30 min, and the absorbance of the resulting color was measured at 450 nm using a spectrophotometer. 

#### 3.3.5. Bipyridine Metal Chelating Activity

The chelating activity of Fe^2+^ ions was analyzed according to the method described by Re et al. [65]. To the test tubes, 125 μL of 2 mM iron sulfate (FeSO_4_) solution was added, followed by the addition of the samples. Then, 500 μL of Tris-HCl buffer solution (pH 7.4) was added, and the mixtures were kept in a dark environment for 30 min. Subsequently, 500 μL of 0.2% bipyridine solution dissolved in 0.2 M HCl was added, followed by the addition of 1250 μL of ethanol and 625 μL of distilled water to complete the reaction. The absorbance values of the samples were measured at 522 nm using a spectrophotometer, with Tris-HCl buffer used as a reference [67].

(5) $$Metal\;Chelating\;Activity\;(Inhibition\;\%)=\frac{A_{control}-A_{sample}}{A_{control}}\times 100$$

A_control_: the absorbance of the control, A_sample_: the absorbance of the sample.

### 3.4. Identification of Phenolic Compounds

The determination of phenolic compounds was carried out using an HPLC-DAD instrument according to the method reported by Küçükaydın et al. [68]. Within the scope of the analysis, injections of phenolic reference compounds were performed, and their retention times were determined to create calibration curves. Based on the obtained data, the samples were evaluated both qualitatively and quantitatively, and the phenolic compound profile was analyzed. The results of phenolic compound analyses were expressed as milligrams of compound per gram of dry weight (mg/g DW) of plant material.

### 3.5. Statistical Analysis

Statistical analyses were conducted using SPSS 20.0 software to evaluate differences among the data obtained from biochemical characterization studies. Data were expressed as mean ± standard error (SE) of three independent replicates (*n* = 3). Prior to analysis, data were tested for normality using the Shapiro–Wilk test and for homogeneity of variances using Levene’s test. As the assumptions of normal distribution and variance homogeneity were satisfied (*p* > 0.05), a parametric approach was considered appropriate. Therefore, one-way ANOVA was performed to compare group means, followed by Duncan’s multiple comparison test as a post hoc procedure to determine significant differences between treatments at *p* < 0.05. When assumptions were not met, appropriate data transformations (logarithmic or square root) were applied before analysis [69]. In the present research, a PCA biplot of antioxidant activity in ethanol and herbal tea extracts was generated to visualize the multivariate distribution and clustering patterns of samples based on assay responses. In addition, a heatmap of correlations between phenolic compounds and antioxidant capacity was produced to illustrate the directional strength of linear associations among variables. All multivariate analyses were conducted in R 3.6.2 (The R Foundation for Statistical Computing, Vienna, Austria) using the relevant statistical packages (‘factoextra’ and ‘ggplot2’).

## 4. Conclusions

This study showed that herbal teas from eight medicinal plants grown in Southwestern Türkiye possessed stronger antioxidant activity than ethanol extracts, with rosemary tea performing best due to its high rosmarinic acid content. Lavender and eucalyptus teas also showed notable potential. The results highlight the strong influence of the extraction method on phenolic composition and support the traditional use of these plants as natural antioxidant sources. Overall, herbal teas appear promising for functional food and nutraceutical applications, and clinical studies are needed to confirm their health effects.

## Figures and Tables

**Table 1:** Antioxidant activity values of ethanol extracts and herbal teas.

		β-Carotene–Linoleic Acid Assay	DPPH^•^ Assay	ABTS^+^ Assay	CUPRAC Assay	Metal Chelating Assay
		IC_50_ (µg/mL)	IC_50_ (µg/mL)	IC_50_ (µg/mL)	A_0.50_ (µg/mL)	IC_50_ (µg/mL)
Ethanol	Rosemary	3.55 ± 0.17a	6.12 ± 0.25a	4.80 ± 0.36a	5.24 ± 0.33a	14.71 ± 0.45a
Herbal Tea		2.90 ± 0.11a	4.76 ± 0.18a	3.42 ± 0.15a	3.88 ± 0.17a	9.22 ± 0.13a
Ethanol	Laurel	18.21 ± 0.31c	27.45 ± 0.36d	21.68 ± 0.56d	28.37 ± 0.14d	42.29 ± 0.35c
Herbal Tea		15.37 ± 0.26c	22.54 ± 0.42c	14.80 ± 0.63c	19.11 ± 0.35c	31.57 ± 0.44b
Ethanol	Lavender	18.27 ± 0.38c	22.98 ± 0.51c	13.59 ± 0.33b	15.68 ± 0.39b	40.69 ± 0.27c
Herbal Tea		11.30 ± 0.24b	17.61 ± 0.64b	10.85 ± 0.27b	13.27 ± 0.20b	35.41 ± 0.71b
Ethanol	Eucalyptus	16.79 ± 0.29c	22.65 ± 0.52c	14.56 ± 0.90c	18.50 ± 0.31c	36.78 ± 0.60b
Herbal Tea		8.97 ± 0.12b	18.10 ± 0.47b	11.05 ± 0.23b	15.66 ± 0.54b	28.78 ± 0.68b
Ethanol	Liquidambar	24.80 ± 0.44d	21.34 ± 0.58c	19.22 ± 0.69c	32.77 ± 0.35d	85.90 ± 0.66d
Herbal Tea		19.97 ± 0.23c	17.82 ± 0.39b	14.56 ± 0.42b	25.08 ± 0.18c	71.30 ± 0.88c
Ethanol	Myrtle	16.19 ± 0.26c	18.21 ± 0.44b	17.54 ± 0.48c	19.60 ± 0.56c	27.67 ± 0.22b
Herbal Tea		12.20 ± 0.14b	16.80 ± 0.38b	10.95 ± 0.27b	15.69 ± 0.40b	18.95 ± 0.31a
Ethanol	Olive	62.78 ± 0.80e	77.56 ± 0.67e	75.09 ± 0.93e	70.28 ± 0.55e	92.80 ± 0.66e
Herbal Tea		51.47 ± 0.95e	65.20 ± 0.84e	61.04 ± 0.58e	58.20 ± 0.63e	81.17 ± 0.73d
Ethanol	Vitex	73.91 ± 0.25e	81.61 ± 0.76e	61.90 ± 0.60e	80.96 ± 0.87e	132.1 ± 0.86e
Herbal Tea		55.36 ± 0.85e	69.94 ± 0.88e	54.23 ± 0.58e	65.80 ± 0.73e	95.72 ± 0.95e
	α-tocopherol	2.10 ± 0.05	38.20 ± 0.50	35.50 ± 0.55	60.20 ± 0.45	-
	BHA	1.50 ± 0.03	19.50 ± 0.30	12.70 ± 0.10	25.40 ± 0.38	-
	EDTA	-	-	-	-	5.50 ± 0.35

Values are means ± SE of three replicates (*n = 3*). Different letters indicate significant differences according to Duncan’s multiple comparison test (*p* < 0.05).

**Table 2:** Phenolic compounds and their strongest correlations with antioxidant assays.

Phenolic Compound	Antioxidant Assay Showing Highest Correlation	Pearson r
Rosmarinic acid	ABTS	0.982
Quercetin	CUPRAC	0.955
Ferulic acid	DPPH	0.912
Caffeic acid	ABTS	0.889
Ellagic acid	Metal chelation	0.721
p-Hydroxybenzoic acid	CUPRAC	0.684
Syringic acid	Metal chelation	0.641

The table summarizes the strongest Pearson correlations (r) observed between individual phenolic compounds and antioxidant activity assays in herbal tea samples.

## Data Availability

Data will be made available on request. In addition, all research data related to this study are securely stored on both an external hard drive and a virtual disk.

## Supplementary Materials

The following supporting information can be downloaded at https://www.mdpi.com/article/10.3390/plants15010125/s1, Table S1: Extraction yields of the extracts; Table S2: Calibration equations, regression coefficients (R^2^), linearity ranges, LODs, LOQs, of phenolic standards at 280 nm; Table S3: Phenolic composition of the extracts by HPLC-DAD (mg/g).
