# Supplementary material for: Biochemical Diversity and Nutraceutical Potential of Medicinal Plant-Based Herbal Teas from Southwestern Türkiye
Source: Plants (Basel). 2026 Jan 1;15(1):125. doi: 10.3390/plants15010125 (PMC12787651; doi:10.3390/plants15010125)
Supplement: Supplementary file 1 [file plants-15-00125-s001.zip › plants-3940953-supplementary.pdf]

Table S1. Extraction yields of the extracts

Solvent	Plant	Extraction Yield (%)
Ethanol	Rosemary	12.35
Herbal tea	Rosemary	10.50
Ethanol	Laurel	11.80
Herbal tea	Laurel	8.25
Ethanol	Lavender	11.75
Herbal tea	Lavender	9.65
Ethanol	Eucalyptus	10.45
Herbal tea	Eucalyptus	8.30
Ethanol	Liquidambar	8.20
Herbal tea	Liquidambar	6.70
Ethanol	Myrtle	10.05
Herbal tea	Myrtle	7.95
Ethanol	Olive	6.25
Herbal tea	Olive	4.80
Ethanol	Vitex	6.75
Herbal tea	Vitex	5.20

Table S2. Calibration equations, regression coefficients (R^2), linearity ranges, LODs, LOQs, of phenolic standards at 280 nm.

No	Compounds	Calibration equation	$R^{2\,a}$	Linear range (µg/mL)	LOD [b] (µg/mL)	LOQ [c] (µg/mL)
**1**	Gallic acid	y = 12540x + 132.6	0.9994	2.00-200	1.28	2.70
**2**	Protocatechuic acid	y = 11897x + 142.5	0.9998	0.45-250	0.30	0.85
**3**	Catechin	y = 11150x + 130.0	0.9992	2.50-180	2.15	3.65
**4**	Pyrocatechol	y = 9800x + 148.0	0.9995	1.70-250	1.56	3.80
**5**	Chlorogenic acid	y = 10970x + 125.9	0.9995	1.80-220	1.45	2.90
**6**	*p*-Hydroxybenzoic acid	y = 10285x + 110.4	0.9994	0.50-190	0.32	0.92
**7**	6,7-Dihydroxy coumarin	y = 10150x + 121.0	0.9993	0.30-250	0.18	0.45
**8**	Caffeic acid	y = 11460x + 129.7	0.9997	0.36-200	0.30	0.84
**9**	3-Hydroxybenzoic acid	y = 10010x + 130.0	0.9991	1.50-120	1.15	3.22
**10**	Syringic acid	y = 10120x + 108.5	0.9995	0.85-150	0.77	2.10
**11**	Vanillin	y = 9750x + 121.0	0.9998	2.20-150	1.95	4.12
**12**	*p*-Coumaric acid	y = 10840x + 120.3	0.9992	2.80-100	2.65	5.25
**13**	Taxifolin	y = 11220x + 130.0	0.9996	1.25-200	0.92	2.15
**14**	Ferulic acid	y = 10350x + 112.1	0.9994	0.70-140	0.58	1.24
**15**	Coumarin	y = 9600x + 155.0	0.9992	1.50-120	0.85	2.42
**16**	Rutin	y = 9875x + 105.6	0.9991	1.44-250	1.15	2.86
**17**	Ellagic acid	y = 9950x + 107.8	0.9990	10-220	6.43	14.70
**18**	Rosmarinic acid	y = 12110x + 144.9	0.9996	0.40-280	0.25	0.72
**19**	Myricetin	y = 10780x + 118.4	0.9998	0.50-250	0.42	0.95
**20**	Quercetin	y = 11250x + 124.6	0.9996	0.75-200	0.60	1.57
**21**	*trans*-Cinnamic acid	y = 10110x + 111.3	0.9998	0.50-250	0.36	1.10
**22**	Luteolin	y = 10485x + 119.0	0.9993	0.80-200	0.55	1.25
**23**	Hesperetin	y = 10020x + 144.0	0.9991	3.15-200	3.10	5.28
**24**	Kaempferol	y = 10030x + 110.4	0.9990	1.20-180	0.90	2.10
**25**	Apigenin	y = 9830x + 106.9	0.9990	3.80-220	3.55	5.74
**26**	Chrysin	y = 9650x + 101.2	0.9995	0.30-150	0.35	0.95

[a] R^2: linearity of the calibration graph, [b] LOD: Limit of Detection in µg/mL, [c] LOQ: Limit of Quantification in µg/mL.

Table S3. Phenolic composition of the extracts by HPLC-DAD (mg/g).

Phenolic compounds	RT (min)	Rosemary (ethanol)	Rosemary (herbal tea)	Laurel (ethanol)	Laurel (herbal tea)	Lavender (ethanol)	Lavender (herbal tea)	Eucalyptus (ethanol)	Eucalyptus (herbal tea)	F Values
Gallic acid	5.70	nd	nd	nd	nd	nd	nd	0.33 d	0.42 d	21.154
Protocatechuic acid	8.75	0.10 e	0.24 de	nd	nd	1.80 d	4.08 c	0.28 de	0.55 de	94.825
Catechin	10.68	nd	nd	nd	0.17 a	nd	nd	nd	nd	5.522
Pyrocatechol	11.04	nd	nd	nd	0.15 b	nd	nd	nd	nd	9.765
Chlorogenic acid	12.35	0.18 e	0.78 d	nd	nd	3.51 b	5.80 a	0.13 e	0.25 e	135.037
p-hydroxy benzoic acid	12.77	0.13 d	0.52 bc	nd	nd	0.74 a	0.68 ab	nd	nd	23.961
6.7-Dihydroxy coumarin	14.10	nd	nd	0.11 b	0.24 a	nd	nd	nd	nd	11.281
Caffeic acid	15.09	0.21 c	0.36 c	0.20 c	0.35 c	8.36 b	13.20 a	0.41 c	0.74 c	211.519
Syringic acid	16.56	nd	0.15 b	0.12 bc	0.27 a	nd	nd	nd	nd	48.118
p-Coumaric acid	20.56	nd	0.11 d	0.05 d	0.12 d	0.37 c	0.51 c	nd	nd	175.488
Ferulic acid	22.14	0.17 efg	0.42 def	0.07 fg	0.19 efg	0.22 efg	0.28 efg	nd	nd	396.443
Coumarin	24.49	nd	nd	nd	nd	nd	nd	nd	nd	23.012
Rutin	25.30	0.11 d	0.23 d	nd	nd	nd	nd	1.05 b	1.38 a	31.613
Ellagic acid	26.11	0.10 b	0.15 b	nd	nd	nd	nd	0.14 b	0.17 b	6.362
Rosmarinic acid	26.77	12.51 bc	30.74 a	nd	nd	7.68 cd	19.25 b	nd	nd	11.896
Myricetin	27.35	nd	nd	nd	nd	nd	nd	3.17 d	5.58 c	136.123
Quercetin	30.83	0.30 h	0.74 gh	7.92 c	13.56 a	0.10 h	0.15 h	1.37 fg	1.63 ef	264.058
trans-cinnamic acid	31.33	0.10 cd	0.29 c	0.10 cd	0.17 cd	0.23 c	0.28 c	nd	nd	264.067
Luteolin	31.70	0.13 c	0.30 c	nd	nd	nd	nd	0.60 c	0.63 c	73.408
Kaempferol	33.21	nd	nd	2.10 d	5.23 a	nd	nd	nd	nd	228.804
Apigenin	33.77	nd	nd	nd	nd	0.16 b	0.25 a	0.05 cd	0.08 c	14.425
Chrysin	38.40	0.16 d	0.28 cd	0.65 b	1.18 a	nd	nd	nd	nd	8.203

Phenolic compounds	RT (min)	Liquidambar (ethanol)	Liquidambar (herbal tea)	Myrtle (ethanol)	Myrtle (herbal tea)	Olive (ethanol)	Olive (herbal tea)	Vitex (ethanol)	Vitex (herbal tea)	F Values
Gallic acid	5.70	3.75 b	5.10 a	1.86 c	3.78 b	nd	nd	0.11 d	0.19 d	21.154

Protocatechuic acid	8.75	1.85 d	3.51 c	0.33 de	0.59 de	nd	nd	11.56 b	17.68 a	94.825
Catechin	10.68	nd	nd	nd	nd	nd	nd	nd	nd	5.522
Pyrocatechol	11.04	0.32 a	0.28 a	nd	nd	nd	nd	nd	nd	9.765
Chlorogenic acid	12.35	nd	nd	nd	nd	nd	nd	0.72 d	1.28 c	135.037
p-hydroxy benzoic acid	12.77	nd	nd	nd	nd	nd	nd	0.36 c	0.45 c	23.961
6.7-Dihydroxy coumarin	14.10	nd	nd	nd	nd	nd	nd	nd	nd	11.281
Caffeic acid	15.09	0.21 c	0.35 c	0.16 c	0.18 c	0.28 c	0.43 c	0.65 c	0.90 c	211.519
Syringic acid	16.56	nd	nd	0.11 c	0.12 bc	nd	nd	nd	nd	48.118
p-Coumaric acid	20.56	2.10 b	3.65 a	nd	nd	nd	nd	nd	nd	175.488
Ferulic acid	22.14	nd	nd	0.68 cd	0.96 c	5.89 b	7.15 a	0.39 def	0.52 de	396.443
Coumarin	24.49	nd	nd	nd	nd	2.20 b	2.71 a	nd	nd	23.012
Rutin	25.30	1.08 b	1.57 a	nd	nd	0.31 d	0.65 c	nd	nd	31.613
Ellagic acid	26.11	nd	nd	2.87 a	4.18 a	nd	nd	nd	nd	6.362
Rosmarinic acid	26.77	nd	nd	nd	nd	1.04 d	1.92 d	nd	nd	11.896
Myricetin	27.35	7.54 b	11.10 a	3.80 d	5.40 c	nd	nd	nd	nd	136.123
Quercetin	30.83	2.25 e	4.60 d	0.13 h	0.18 h	7.32 c	10.60 b	0.10 h	0.11 h	264.058
trans-cinnamic acid	31.33	1.90 b	3.93 a	nd	nd	nd	nd	nd	nd	264.067
Luteolin	31.70	nd	nd	nd	nd	2.70 b	4.02 a	3.84 a	4.40 a	73.408
Kaempferol	33.21	nd	nd	2.80 c	3.64 b	nd	nd	nd	nd	228.804
Apigenin	33.77	nd	nd	nd	nd	nd	nd	nd	nd	14.425
Chrysin	38.40	nd	nd	nd	nd	nd	nd	0.37 bcd	0.55 bc	8.203

Values are means ± SE of three replicates (n = 3). Different letters indicate significant differences according to Duncan's multiple comparison test ($p < 0.05$)./ nd: not detected.
